# “Multisystem Inflammatory Syndrome in Children” (MIS-C) after COVID-19 Infection in the Metropolitan Area of Nuremberg-Erlangen, Germany—Expectations and Results of a Two-Year Period

**DOI:** 10.3390/children10081363

**Published:** 2023-08-09

**Authors:** Steven Hébert, Marius Schmidt, Georg Topf, Daniel Rieger, Jens Klinge, Jan Vermehren, Christoph Fusch, Christian Grillhösl, Michael Schroth, Irmgard Toni, Heiko Reutter, Patrick Morhart, Gregor Hanslik, Linda Mulzer, Joachim Woelfle, Bettina Hohberger, André Hoerning

**Affiliations:** 1 Department of Pediatrics and Adolescent Medicine, University Hospital Erlangen, 91054 Erlangen, Germany; 2 Hospital for Children and Adolescents Furth, 90766 Furth, Germany; 3 Pediatrics—Children’s Department Nuremberg Hospital South, 90471 Nuremberg, Germany; 4 Cnopf Children’s Hospital, Diakoneo Klinikum Hallerwiese Nuremberg, 90419 Nuremberg, Germany; 5 Department of Ophthalmology, University Hospital Erlangen, 90766 Erlangen, Germany

**Keywords:** IVIG, intravenous immunoglobulins, MIS-C, Multisystem Inflammatory Syndrome in children, mAbs, monoclonal antibodies, COVID-19

## Abstract

Background: Multisystemic Inflammatory Syndrome in children (MIS-C) is a rare autoimmune disorder occurring after a latency period following acute SARS-CoV-2 infection. The therapeutic regime of MIS-C is adapted to the therapy of the Kawasaki disease, as clinical symptoms are similar. Since the Kawasaki disease can potentially result in severe symptoms, which may even affect long-term health, it is essential to gain further knowledge about MIS-C. Thus, we aimed to investigate the incidence, symptoms, therapeutical procedure and outcome of MIS-C patients in the metropolitan area of Nuremberg-Erlangen during the SARS-CoV2 pandemic. Material and Methods: Retrospective analysis of clinical charts of MIS-C patients was carried out at three children’s hospitals covering the medical care of the metropolitan area of Nuremberg-Erlangen in Germany. Demographic characteristics and symptoms at first visit, their clinical course, therapeutic regime and outcome were recorded within the time period January 2021–December 2022. Results: Analysis of 10 patients (5 male, 5 female) with MIS-C resulting in an incidence of 2.14/100.000 children. The median time between COVID-19 infection and admission to hospital was 5 weeks. The median age was 7 years. Symptoms comprised fever (100%), rash (70%), bilateral non-purulent conjunctivitis (70%) and urticaria (20%). At the time of presentation, diagnosis-defining inflammation parameters were increased and the range for C-reactive protein was 4.13 mg/dL to 28 mg/dL, with a median of 24.7 mg/dL. Procalcitonin was initially determined in six patients (1.92 ng/mL to 21.5 ng/mL) with a median value of 5.5 pg/mL. Two patients displayed leukocytosis and two displayed leukopenia. None of the patients presented coronary pathologies. Nine of the ten patients received intravenous immunoglobulin (IVIG) therapy. In addition, patients received intravenous steroids (80%) and acetylsalicylic acid (80%). Conclusion: SARS-CoV virus may rarely exert multiorgan manifestations due to hyperinflammatory immunological processes. Within two years of the COVID-19 pandemic, we identified ten patients with COVID-induced MIS-C in the metropolitan area Nuremberg-Erlangen. In the description of the patient collective, we can confirm that MIS-C is distinguished from the Kawasaki disease by the lack of coronary manifestations. Interestingly, although having monitored all pediatric facilities in the investigated area, we find lower incidences of MIS-C compared to findings in the literature. In conclusion, an overestimation of incidences in the upcoming MIS-C during the pandemic needs to be considered.

## 1. Introduction

The worldwide pandemic with the SARS-CoV-2 virus has occupied medical resources and has appeared in severe and high mortality disease courses. Some of these required intensive care treatment, especially in adult medicine. In the majority of cases, children showed rather mild symptoms when dealing with acute infection with SARS-CoV-2 [1]. However, children, more than adults, seem to be affected by a dysregulation of the immune system, appearing weeks after the primary infection [2]. Those symptoms were soon to be described as “Multisystem Inflammatory Syndrome in children” (MIS-C) [3]. This led the Royal College of Pediatrics and Child Health and, subsequently, the World Health Organization (WHO) to release a case definition of MIS-C in 2020 [4]. Core elements of MIS-C are similar but not identical to the Kawasaki disease. Persistent fever combined with a heterogeneous field of skin manifestations, cardiovascular, gastrointestinal and hematological symptoms and elevated inflammation markers with evidence of single or multi-organ dysfunction (shock, cardiac, respiratory, renal, gastrointestinal or neurological disorder) were described [5]. Any microbial cause, including bacterial sepsis, streptococcal shock syndromes, other bacterial sepsis, as well as infections associated with myocarditis, need to be primarily ruled out. The exact pathophysiology still needs more research to be fully understood. However, findings show that a post-infectious overreaction of the immune system, e.g., following a viral infection, leads to an increase of proinflammatory cytokines, eventually culminating in a so-called “cytokine storm”. This causes, among others, vasculitis and endothelial damage [6]. After infection with SARS-CoV-2, the immune system is activated to fight off the virus. In most cases, the immune response is well-coordinated and helps to control the infection. However, in some individuals, the immune response becomes dysregulated, leading to the release of excessive amounts of pro-inflammatory cytokines. The cytokine storm in COVID-19 is thought to be either triggered by the virus directly or by the immune system’s response to the virus. The excessive release of cytokines can result in widespread inflammation and damage to various organs and tissue throughout the body. This immune overreaction can contribute to the severity of the disease and potentially lead to complications.

Here, we investigated the frequency, cause and description of children that developed MIS-C following a SARS-CoV-2 infection within the metropolitan area of Nuremberg-Erlangen during the pandemic. The aim of this study was to critically investigate whether the regional data is consistent with other publications. We compared the data to existing literature published in the early phase of the COVID-19 pandemic in order to rule out any bias of early pandemic overrating of clinical conditions in our pediatric patient collective.

## 2. Patients and Methods

We performed a retrospective analysis of digital and paper-based clinical charts within the period of the first two years of the pandemic, from January 2020 until December 2022. We gathered all information systematically in an Excel data sheet and evaluated this data by descriptive statistical methods. All participating centers received a table matrix with specific criteria of MIS-C for documentation. We investigated the aspects of the data regarding the distribution numerically, in percentage, median and average. Results are described by text and are presented in graphs and in a supplementary chart. A total of 10 pediatric patients with Multisystemic Inflammatory Syndrome were included in this study. All patients had to fulfill the required criteria of the WHO case definition of MIS-C to be considered eligible. Patient data was collected retrospectively and anonymously from the corresponding documentation systems of the four participating hospitals for children and adolescents in Germany: Department of Pediatrics and Adolescent Medicine, Friedrich–Alexander University Erlangen, Erlangen, Hospital for Children and Adolescents in Furth, Children’s Department Nuremberg Hospital South and Cnopf’sche Children´s Clinic/Hallerwiese in Nuremberg, representing the complete inpatient medical care within the metropolitan area of Nuremberg- Erlangen. This investigated area comprises 3.6 million inhabitants and consists of a statistical total of 13% children. According to the respected case definition of the WHO, we collected data on clinical symptoms (skin/conjunctivitis/mucocutaneous manifestation, cardiac, hematological and gastrointestinal, fever, inflammation markers and liver/kidney parameters), as well as information about previous SARS-CoV-2 infection. Additionally, information about epidemiology, time of SARS-CoV-2 infection/antibody status and treatment were collected. The data of 10 patients with previous SARS-CoV-2 infection is shown in the supplementary file Table S1.

### Cardiomyocyte Bioassay

An established sensitive cardiomyocyte-bioassay was used for the measurement of the GPCR-AAb in the case of one patient. The experiments are previously described in detail by Wallukat et al. Briefly, the beating rate of rat cardiomyocytes, which were cultured for 4 days, were counted on 6 selected fields of the culture flask placed on a heated stage of an inverted microscope (37 °C, for 15 s). The cut-off was 1.8 beats/15 s.

## 3. Results

### 3.1. Demographic Data

**Figure 1:**
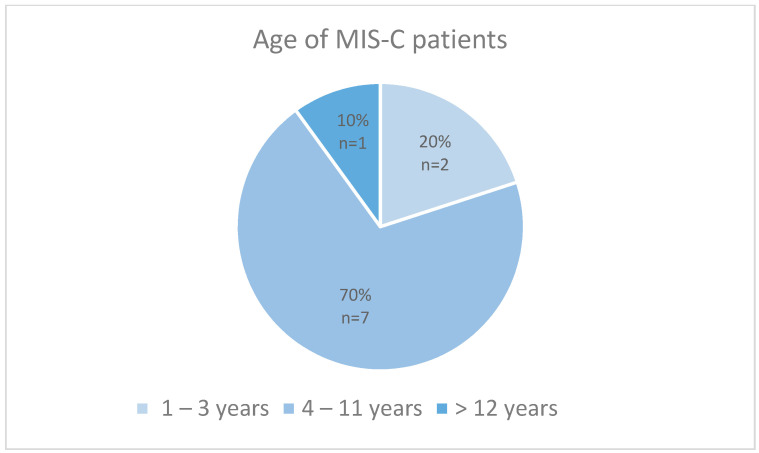
Distribution of age in the MIS-C patient collective.

**Figure 2:**
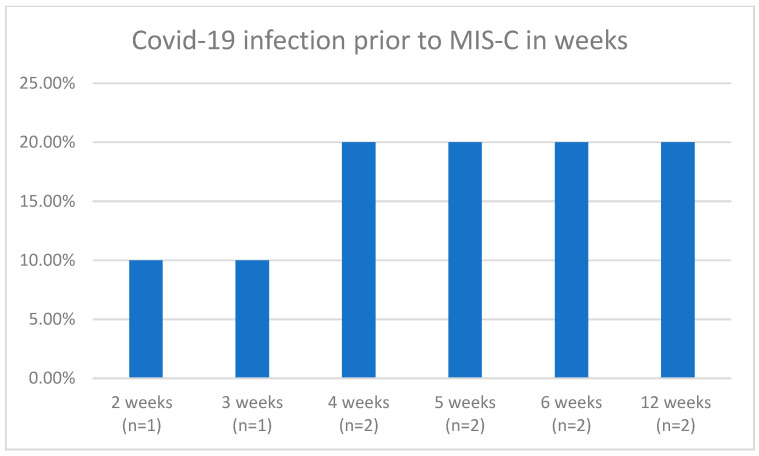
COVID-19 infection prior to onset MIS-C in weeks (y-axis: amount of patients in percentage [10% = 1 patient]; x-axis: number of weeks of COVID-19 infection prior to onset MIS-C).

Within the metropolitan area of Nuremberg-Erlangen, 10 children were documented with a Multisystemic Inflammatory Disease following the COVID-19 disease. We considered all patients under the age of 18 years. The age ranged from 1 year to 14 years, with a median age of 7 years (Figure 1). Out of all the participants, 50% (*n* = 5) were female and a total of 8 patients (80%) had no comorbidities and were considered as healthy individuals upon admission. One patient presented with a comorbidity of a BMI > 90 P. and another patient presented with a hemodynamic stable ventricular septum defect. The investigated cohort developed MIS-C after a median of 5 weeks after a confirmed COVID-19 infection (Figure 2).

### 3.2. Symptoms and Laboratory Constellation at First Visit

**Figure 3:**
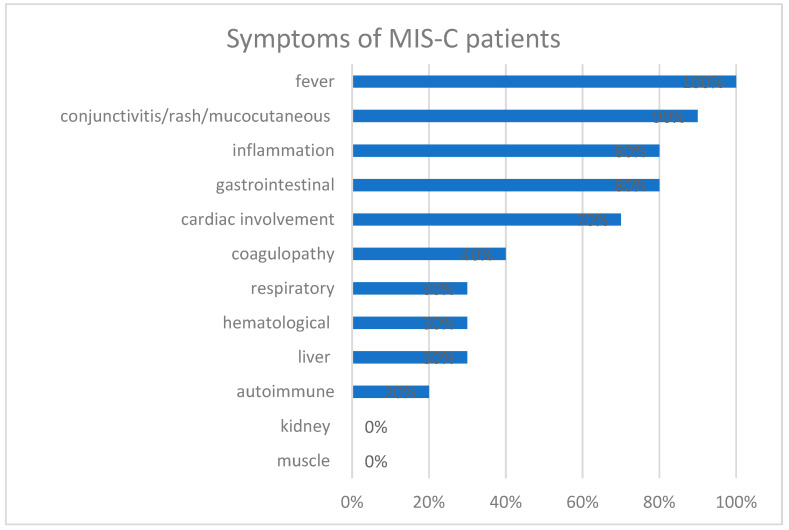
Distribution of the number of MIS-C patients with a minimum of one (or more) symptoms per symptom category in percentage (*n* = 10; 100%).

Patients’ symptoms at the first visit were fever in the case of all patients (100%). A total of 50% (*n* = 5) of the collective presented with bilateral non-purulent conjunctivitis. Another 60% (*n* = 6) presented a rash, and one-third of these children (*n* = 2) suffered from urticaria. Cardiac involvement was stated on the premises of clinical presentation and laboratory values. Pro-BNP ranged from 817 pg/mL to >35,000 pg/mL. Additionally, 50% (*n* = 5) showed pro-BNP levels over 5400 pg/mL. As far as there are general cut-offs for pro-BNP in particular age groups, our laboratory stated normal ranges from 37–1000 pg/mL for ages 1 month to 11 months of age, 39–675 pg/mL for the age 12 months to 35 months, 23–327 pg/mL for the ages 3 years to 6 years and 10–242 pg/mL for the age of 7 years to 14 years. Most of these patients (*n* = 4) had clinical manifestation by AV Block I or II, a spiking of T-waves and, in one of the cases, a pro-BNP >35,000 pg/mL with a severe reduction of cardiac contractility, most likely resulting from catecholamine therapy. Troponin was not evaluated in all cases; however, the results present a value of over 49.5 ng/L in patients (*n* = 2), who were also suffering from clinical symptoms. The reference for our laboratory-specific cut-off was 5 ng/L. None of the patients presented any clinical symptoms of coronary pathologies. Three patients (30%) measured a thrombocytosis, with max. 841 × 10³/µL and ranging elevated D-Dimers of 4.45 mg/L to max. 17.8 mg/L. None of the patients presented a severe infringement of coagulation. Concerning gastrointestinal symptoms, 60% (*n* = 6) of patients presented with diarrhea, 50% (*n* = 5) with abdominal pain, 30% (*n* = 3) with vomiting and two children (20%) with ascites. Only one patient showed no gastrointestinal symptoms. Liver enzymes, Bilirubin and Creatinine were taken from all patients to monitor liver and kidney function, showing a moderate increase in values in only a few patients, with no clinical relevance and normalization in all patients upon recovery. To estimate laboratory signs of infection, a C-reactive protein was taken from every patient, ranging from 4.13 mg/dL to 28 mg/dL, with a median CRP of 24.7 mg/dL (normal range < 5 mg/dL). PCT was evaluated in 60% of all patients, showing values from 1.92 ng/mL to 21.5 ng/mL with a median value of 5.5 ng/mL (normal range < 0.1 ng/mL). In addition, 20% (*n* = 2) of the patients presented leukocytosis, and two patients (20%) presented leukopenia (Figure 3). Figure 3 presents the distribution of symptoms (e.g., fever), as well as related symptom categories, as they are defined by the WHO—classification for MIS-C—and as they are accordingly presented in the supplementary Table S1. In the case of one patient, we measured functional autoantibodies against the G-protein-coupled receptors with a bioassay of spontaneously beating rat cardiomyocytes in an external laboratory. Here we gained positive results for the existence of autoantibodies against Beta2-adrenoreceptors, Muscarinic-M2-receptors and Angiotensin-II-AT1-receptors. Six months after curing the patient, the same examination proved to be negative for the existence of all investigated autoantibodies.

### 3.3. Treatment of MIS-C Patients

All patients received intravenous immunoglobulin (IVIG) at least once, and 20% of all patients received IVIG twice. A total of 2 g/kg/bodyweight IVIG was administered in 90% (*n* = 8) of all patients, and only one patient received 1 g/kg bodyweight as an initial dosage. Those receiving it twice were given a second dosage of 2 g/kg/bodyweight. In one particular case, the patient received 1.33 g /kg/bodyweight. Simultaneously, 80% of all patients received steroids, either prednisolone (2 mg/kg bodyweight) in 70% (*n* = 7) or, in the case of one patient, methylprednisolone in an unknown dosage. All patients receiving prednisolone were treated for 4–6 weeks with corresponding reduction plans. The patient treated with methylprednisolone was treated for three days with an intravenous injection followed by an additional three days with an oral dosage of 2 mg/kg/bodyweight for each application. Acetylsalicylic acid was administered to 40% (*n* = 4) of all patients. One patient received ASS in a dosage of 30 mg/kg/bodyweight daily for three days, following 5 mg/kg/bodyweight daily and continuing beyond dismissal. Two patients received ASS in a dosage of 3 mg/kg/bodyweight and one patient received 4 mg/kg/bodyweight daily, also continuing beyond dismissal.

### 3.4. Outcome of MIS-C Patients

All investigated MIS-C patients were followed up in the period of 23 January to 23 May. There was no loss in follow-up and all patients fortunately fully recovered in restitutio ad integrum based on clinical examinations and lab chemistry parameters. In the case of the patient where autoantibodies against G-protein-coupled receptors were tested, results proved to be negative six months after the MIS-C diagnosis. Practically, this meant that no autoantibodies could be detected in a second testing after treatment and curing. 

## 4. Discussion

Acute infection with the SARS-CoV-2 virus primarily affects the respiratory system. Apart from this manifestation, several other organs, e.g., the heart, kidney, small intestine and the vascular system, can be attacked [7,8]. Interestingly, in children, SARS-CoV-2 generally tends to have rather mild symptoms [9]. Additionally, mild and self-limiting impairments of the liver during the SARS-CoV-2 infection in children have been described [10]. In contrast, MIS-C is a severe and rare condition, affecting children and adolescents with an association to the SARS-CoV-2 virus. It is characterized by widespread inflammation and involvement of various described organ systems [6]. Symptoms of MIS-C include fever, rash, joint pain and swelling, stomach pain, vomiting and heart insufficiency. The clinical characteristics of MIS-C are very similar to the Kawasaki disease. The Kawasaki disease is a medium-vessel vasculitis which particularly affects coronary arteries. Clinically, it is apparent with lymph node swelling, mucocutaneous inflammation (dry, red cracked lips; swollen, bumpy, red tongue) and a polymorphous rash. According to Singh et al., it can affect the liver, lungs, the gastrointestinal system and the central nervous system. Aneurysm of the coronary arteries is a dreaded complication. Elevated inflammation markers and altered blood count are also part of the diagnosis [11]. Based on Nakra et al., MIS-C, compared to the Kawasaki disease, shows various symptoms, e.g., reduced vigilance, gastrointestinal and pulmonal afflictions as well as hypotension. Similar to MIS-C, IVIG is a central part of the therapy [12]. Several key differences in the lab values can roughly differentiate between MIS-C and Kawasaki. For instance, the C-reactive protein, which is produced by the liver in response to inflammation, can be typically elevated in both KD and MIS-C. However, the degree of elevation may be higher in MIS-C than in KD. In KD, CRP levels are typically greater than 3 mg/dL, which is considered significantly elevated [13]. In MIS-C, CRP levels are typically very high, often exceeding 10 mg/dL [14]. Erythrocyte sedimentation rate (ESR) levels are also typically elevated in both KD and MIS-C. In KD, ESR levels are typically greater than 40 mm/h [15]. In MIS-C, ESR levels are also elevated, but may not be as high as in KD. In both KD and MIS-C, white blood cell count (WBC) may be elevated, but it may be higher in MIS-C. In KD, the WBC count is typically between 10,000 and 20,000 cells/mm^3^ [16]. In MIS-C, the WBC count may be significantly higher, often exceeding 20,000 cells/mm^3^. In both KD and MIS-C, platelet count may be low. In KD, platelet count may be less than 150,000 cells/mm^3^ [16]. In MIS-C, platelet count may be significantly lower, often less than 100,000 cells/mm^3^ [14]. However, laboratory values alone are not sufficient to diagnose Kawasaki disease or MIS-C. Additionally, it seems apparent that laboratory values alone are in the same way insufficient to distinguish the two diseases from each other. Both conditions are diagnosed based on a combination of clinical symptoms, laboratory findings and medical history. 

However, MIS-C seems to present as a more severe condition that can cause long-term health problems and is considered to be life-threatening in some cases. Additionally, MIS-C is believed to be linked with SARS-CoV-2, whereas, for the Kawasaki disease, no specific viral infection has been identified so far [16,17]. Nevertheless, we can confirm one important difference that seems to reliably distinguish MIS-C from the KD. MIS-C, other than KD, is not associated with coronary complications. This observation of the patients in this study is consistent with findings in other publications. 

The aim of the present study was also to investigate the incidence of MIS-C patients in the metropolitan area of Nuremberg-Erlangen, comprising 3.6 million habitants during the SARS-CoV2 pandemic. 

Statistically, Germany consists of 13% of children in the overall population. Therefore, the investigated area includes a total of approximately 468.000 children. This calculates an incidence for MIS-C in children following a SARS-CoV2-infection in the respected investigated area of 2.14/100.000 children. Surprisingly, however, this is inconsistent with the existing literature. There are several publications indicating incidences of up to 10.3/100.000 children or more [18]. The present study includes all inpatient care facilities for children in the investigated area. Therefore, it seems highly unlikely that MIS-C patients living in this area were diagnosed and treated without being stationary in one of the participating hospitals. Critically, one must at least consider that MIS-C might have been overestimated in the initial phase of the pandemic. If so, we need to reconsider if and why all documented patients in publications with significantly higher incidences factually fulfilled all the criteria for MIS-C. One aspect could be that other infections (e.g., Adenovirus) were initially not ruled out, leading to a misinterpretation of the clinical symptoms. This could hypothetically have resulted in the overdiagnosis of patients declared to have MIS-C. Furthermore, it could be of interest to see if all previously documented cases were clearly distinguished from other autoimmune reactions, such as the KD. We concede, that even if the bias remains, MIS-C patients could have likewise been factual and falsely diagnosed in the designated area.

The exact cause and pathophysiology of MIS-C is still elusive, yet it is believed to be related to an overactive immune response in children who have been infected with SARS-CoV-2 or who have been in close contact with someone suffering from COVID-19 [12].

Treatment for MIS-C typically involves supportive care and the use of immunoglobulin and corticosteroids to reduce inflammation [19]. In severe cases, children may require hospitalization and additional treatments, such as oxygen therapy or intravenous supportive medical therapy [6]. In most cases, in our collective, the patients received the stated therapies. However, the dosage and substances used were not consistent for every patient (Table S1).

In order to better understand the pathophysiology and treatment options of MIS-C patients, we searched for autoantibodies in the case of one of the reported patients. Interestingly these results proved to be positive. The idea for testing was raised by recent reports, which could be worth to be considered in the context of MIS-C. 

Recent studies demonstrated that the SARS-CoV-2 virus infection might induce the development of a variety of autoantibodies (AAbs) of unknown pathophysiological significance. There is evidence that AAbs are generated even during the acute SARS-CoV-2 infection and thus linked to severe cases of COVID-19. These AAbs were observed to target diverse structures, e.g., phospholipids, nuclear antigens, interferon and interleukin-1 receptor antagonist [20]. It is assumed that the AAbs can act as a pathogen, offering the use of immune suppressive therapy even during acute and severe COVID-19. Acute SARS-CoV-2 infection can be characterized by severe vascular damage with endothelial dysfunction or even damage, neutrophil extracellular traps (NET) formation, hypercoagulation, amyloid fibrin microclotting and immunothrombosis. These vascular and autoimmune hypotheses during an acute COVID-19 stage might share overlaps. Endothelial damage induces fibrin formation. Thus, there is a link to the amyloid fibrin microclotting phenomena. In addition, each cellular damage induces a physiological immune response. Yet, it can be assumed that autoimmune phenomena can arise, triggered by cofactors or a genetic predisposition. Exemplarily, anti-IL-1Ra AAbs were observed in patients with MIS-C, inducing the IL-1β-pathways. Next to these AAb, several other AAbs might interplay in this context, potentially being additionally responsible for long-term effects. The observed GPCR-AAb, measured in one of the presented patients, could potentially contribute to this complex molecular pathogenic network with predominant long-term effects, as observed in patients after COVID-19 infection.

The special pathogenic effect of these GPCR-AAb is that they are active in their targets with a prolonged desensibilization, compared to the physiological ligands, respectively. It is assumed that the non-physiological binding characteristics of these GCPR-fAAb disturb the physiological intercellular homeostasis. If these molecular alterations exceed a certain level, clinical symptoms will occur in the patients. This pathogenic aspect was eponymous: functional AAbs targeting GPCR (GPCR-fAAb). This GPCR-fAAb seemed to be present in diverse human fluids. There is evidence that these GPCR-fAAbs are involved in the pathogenesis of several disorders: a link of GPCR-fAAb to Grave’s disease (Morbus Basedow) or cardiomyopathy has already been observed (see review Becker et al.). These GPCR-fAAb typically bind to extracellular loops (e.g., Grave’s disease: GPCR-fAAb targeting the N-terminal epitope of the TSH receptor). Even in the context of SARS-CoV-2, the presence of GCPR-fAAb in sera of patients with long COVID symptoms has been observed, targeting vasoactive receptors [21,22]. Research data, offering a link of GPCR-fAAb to vascular perfusion, were the basis for a successful healing attempt, neutralizing the GPCR-fAAb in a patient with glaucoma and Post-COVID. Recent data support the idea of an involvement of GPCR-fAAb in the dysregulation of microcirculation in patients with Post-COVID. 

The generation of GCPR-fAAb even during acute SARS-CoV-2 infection can be hypothesized considering a molecular mimicry in analogy to Chagas’ cardiomyopathy (β1-fAAb and an infection with Trypanosoma cruzi). The binding of these GCPR-fAAb to their targets is supposed to be dependent on the surrounding of the receptors. In healthy tissue, GPCR-fAAb did not show any effect, neither in ischemic or inflammatory ones. Thus, it can be hypothesized that GPCR-fAAb binds their specific receptor in ischemic or inflammatory regions, as acute COVID-19 infection is known to trigger e.g., microclots, endothelial inflammatory disruptions with consecutive impaired microcirculation, as mentioned earlier [23,24,25]. This vicious cycle might be sustained by a level of circulating GPCR-fAAbs. The disruption of the binding of GPCR-fAAb to their receptors might be a therapeutic approach in disorders with GPCR-fAAb, using an aptamer or unspecific immunoadsorption in therapy refractory cases. IVIG, being the standard therapy for MIS-C, are hypothesized to modulate the immune system and, thus, the autoimmune system, when administered in a relevant dosage. The exact molecular mechanism of IVIG is unknown. Yet, it is assumed that feedback loops, cellular Fcγ receptors and Fc glycosylation mediated the IVIG effects on AAbs. In the case of the positive tested patient with verification of autoantibodies, we were able to show that diagnostics six months after curing showed no detectable autoantibodies. Despite the limitation of the significance in one single patient of our study, it remains consistent with the hypothesis described [26,27,28].

However, even though strong efforts have been undertaken, the pathogenesis of the dysregulation of the immune response in MIS-C still remains unknown. Although substantial progress has been made in defining the features of immune dysregulation in MIS-C, the findings and causal possibilities that have been assumed for the immunopathogenesis of MIS-C remain broad [29]. For instance, SARS-CoV2 (super)antigen persistence, immune cellular abnormalities (decreased plasmacytoid dendritic cells, decreased but highly activated CD4+ and CD8+ T cells), including the development of autoantibodies or immune complexes and a genetic predisposition for autoimmunity, are discussed [30,31].

The long-term outcomes of MIS-C can vary depending on the severity of the condition and the individual patient. Early recognition, prompt medical intervention, and appropriate treatment can significantly improve outcomes. Most children with MIS-C require hospitalization and intensive care support. While the majority of children with MIS-C recover with appropriate treatment, it is important to note that some severe cases can be life-threatening. Long-term outcomes and complications of MIS-C are still being studied and ongoing research aims to better understand the potential long-term effects and outcomes in these patients. It is important for children who have had MIS-C to receive appropriate follow-up care to monitor their health and address any potential long-term consequences. The overall outcome of our investigated patients demonstrated that the established treatment strategies prove to be very effective, as all patients survived and regained complete health. This also confirms the high survival rate in existing literature with approximately only 0.02% death rates in affected children with MIS-C [32]. Interestingly the exact dosage of administered medication in our cohort seems to have no effect on the overall outcome. This ultimately raises the question concerning the therapeutic width of the used substance groups and particular dosages for clinical practice. We acknowledge that there is some limitation in making a statement in this matter considering the number of patients in this study. However, it remains worth mentioning, that the outcome is identical despite some differences in therapeutic approaches between clinics involved in this study.

## 5. Conclusions

In summary, in rare cases, acute infection with the SARS-CoV virus can appear with multiorgan manifestation due to hyperinflammatory immunological processes, called Multisystemic Inflammatory Syndrome in children (MIS-C). Our investigated patients fulfilled the typical criteria for the diagnosis of this autoimmune response mechanism. 

Although MIS-C associated with COVID-19 is often compared to the Kawasaki Disease, it presents with more or less significantly different criteria and both should be clearly distinguished. Particularly the existence of coronary pathologies must raise a strong awareness for KD and argues against MIS-C. Immediate standardized treatment led to complete recovery in all the observed MIS-C patients. A therapeutical approach and monitoring of treatment may be the identification of pathophysiological relevant biomarkers of the exacerbated immune response enabling a standardized determination e.g., of functional active autoantibodies. However, their occurrence and pathophysiological role need to be proven in larger patient cohorts employing adequate age- and sex-matched control subjects. 

In our study, we attained lower incidences of MIS-C compared to a variety of existing literature. Therefore, we suggest considering that, in the flashing events of the first phase of the COVID-19 pandemic, incidences of MIS-C might have been overestimated. It must be examined critically if all documented patients in the past had doubtlessly fulfilled all criteria for MIS-C when diagnosed.

Overall, this regional study emphasizes that even in seemingly very good investigated diseases such as the association of MIS-C to the SARS-CoV2 pandemic, with indisputable global interest, there are still inconsistencies in descriptive aspects. Distinguishing MIS-C from other autoimmune reactions remains an important aspect. 

Finally, we suggest that the possibilities of autoantibodies should be further investigated as they might have the potential to be a sufficient method for diagnostics and treatment for our MIS-C patients.

## Data Availability

The data presented in this study are available on request from the corresponding author, as anonymously documented data sets from clinical data systems of all participating hospitals. The data are not publicly available due to ethical restrictions on the personal data of patients.

## Supplementary Materials

The following supporting information can be downloaded at: https://www.mdpi.com/article/10.3390/children10081363/s1, Table S1: The data of 10 patients with MIS-C after SARS-CoV-2 infection.

## Abbreviations

IVIG, intravenous immunoglobulins; RBCs, Red blood cells; MIS-C, Multisystem Inflammatory Syndrome in children; AST, aspartate transaminase; ALT, alanine transaminase; GGT, gamma-glutamyltransferase; LDH, Lactate dehydrogenase; PT, Prothrombin time; PTT, Partial thromboplastin time; TNF-alpha, TNF-alpha Tumor necrosis factor; IL-2 receptor, interleukin-2 receptor; IL-18, interleukin 18; ANAs, Anti-nuclear antibodies; ACE2, angiotensin-converting enzyme 2; ANAs, anti-nuclear autoantibodies; mAbs, monoclonal antibodies.
